# Educational needs in neuro-oncology: Insights from the European Society for Medical Oncology Central Nervous System Faculty Survey

**DOI:** 10.1093/noajnl/vdag097

**Published:** 2026-05-11

**Authors:** Oriol Mirallas, Giulia Cerretti, Anna Sophie Berghoff, Dieta Brandsma, Filip De Vos, Julia Furtner, Norbert Galldiks, Giuseppe Lombardi, Slavka Lukacova, Evangelia Razis, Ghazaleh Tabatabai, Emilie Le Rhun, Enrico Franceschi, Giuseppe Minniti

**Affiliations:** Medical Oncology Department, Vall d’Hebron Hospital Campus and Vall d’Hebron Institute of Oncology (VHIO), Passeig de la Vall d’Hebron, 119-129, Barcelona 08035, Spain (O.M.); Department of Surgery, Oncology and Gastroenterology, University of Padua, Padua, Italy (G.C.); Division of Oncology / Department of Medicine 1, Medical University of Vienna (A.S.B.); Department of Neurology, Netherlands Cancer Institute, Amsterdam, The Netherlands (D.B.); Department of Medical Oncology, University Medical Center Utrecht, Utrecht, The Netherlands (F.D.V.); Research Center for Medical Image Analysis and Artificial Intelligence (MIAAI), Faculty of Medicine and Dentistry, Danube Private University, Krems, Austria (J.F.); Dept. of Neurology, Faculty of Medicine and University Hospital Cologne, Cologne, Germany; Inst. of Neuroscience and Medicine (INM-3), Research Center Juelich, Juelich, Germany; and Center of Integrated Oncology Aachen Bonn Cologne Duesseldorf (CIO ABCD), Germany (N.G.); Department of Medical Oncology, Oncology 1, Veneto Institute of Oncology IOV-IRCCS, Padua, Italy (G.L.); Department of Oncology, Department of Clinical Medicine, Aarhus University & Aarhus University Hospital, Aarhus, Denmark (S.L.); 3rd oncology department, Hygeia Hospital, Athens, Greece (E.R., E.L.R.); Department of Neurology & Interdisciplinary Neuro-Oncology, University Hospital Tübingen, Hertie Institute for Clinical Brain Research, Center for Neuro-Oncology, Comprehensive Cancer Center Tübingen-Stuttgart, Eberhard Karls University Tübingen, Germany (G.T.); Department of Medical Oncology and Hematology, University Hospital Zurich, Zurich, Switzerland (E.L.R); Nervous System Medical Oncology Department, IRCCS Istituto delle Scienze Neurologiche di Bologna, Bologna, Italy (E.F.); Department of Radiological Sciences, Oncology and Anatomical Pathology, Sapienza University of Rome, Policlinico Umberto I, Rome, Italy; IRCCS Neuromed, Pozzilli IS, Italy (G.M.)

**Keywords:** clinical research training, CNS tumors, molecular diagnostics, neuro-oncology education, supportive care

## Abstract

**Background:**

Primary central nervous system (CNS) tumors are rare malignancies with limited treatment options, resulting in high mortality. Unlike other solid tumors, CNS tumors present unique challenges that impact the health-related quality of life (HRQoL) of patients and caregivers. Effective management requires balancing oncologic treatment with the control of neurocognitive deficits, epilepsy, and other neurological symptoms.

**Methods:**

The European Society for Medical Oncology (ESMO) CNS Faculty conducted a consensus process to identify the most pressing educational priorities for physicians managing CNS malignancies. This process involved structured data collection and expert input to determine critical areas for disease-specific education and training.

**Results:**

The consensus process highlighted key educational gaps in managing CNS tumors, emphasizing the need for a multidisciplinary approach to optimize therapeutic strategies, improve survival outcomes, enhance HRQoL during treatment and survivorship, and facilitate access to emerging therapies. Physicians reported that comprehensive, disease-specific education is essential to address these challenges effectively.

**Conclusion:**

CNS tumors require targeted educational initiatives to equip healthcare providers with the knowledge and skills necessary for optimal patient care. The ESMO CNS Faculty consensus underscores the importance of structured, disease-specific training to improve outcomes, support HRQoL, and advance oncology education in the management of CNS malignancies.

Key Points

- Molecular diagnostics was ranked as the top educational priority among CNS oncology professionals.
- Clinical research design and trial skills were key training gaps identified by survey respondents.
- Multidisciplinary care was rated essential for improving CNS tumor management.
- Supportive and palliative care were highlighted as critical yet under-addressed areas.
- Standardized CNS-focused training and guideline access were widely requested by physicians.

Importance of the Study This study identifies and prioritizes the educational needs of physicians managing primary central nervous system (CNS) tumors, a rare and complex group of malignancies with limited treatment options. By surveying an international cohort of neuro-oncology professionals, it highlights critical gaps in molecular diagnostics, clinical research and trial design, multidisciplinary care, supportive and palliative care, and access to standardized CNS-focused training. Compared with prior literature, this work provides a structured, consensus-based overview of the skills and knowledge most valued by practitioners, emphasizing the practical challenges of translating advances in molecular classification and therapeutic innovation into clinical care. The findings have immediate implications for curriculum development, professional training programs, and international collaboration, supporting targeted educational interventions to improve patient outcomes, optimize clinical decision-making, and facilitate equitable access to emerging therapies in CNS oncology.

Primary tumors of the central nervous system (CNS) account for 1.9% of all cancers globally, ranking 19th in frequency, and causing 2.5% of cancer-related deaths, placing them 12th among the leading causes of cancer mortality. In Europe, incidence rates are 5.6-7.9 per 100 000 with corresponding mortality rates of 3.5 and 5.4 per 100 000 women and men, respectively.^1^

The World Health Organization (WHO) classification of malignant CNS tumors was historically solely based on histological characteristics, meaning morphology and immunohistochemical traits. However, advancements in molecular biomarkers have significantly improved our ability to classify tumor subtypes accurately, facilitating more precise diagnoses and thus the treatment and prognosis of new tumor entities and subtypes. In this context, the WHO 2021 classification made substantial revisions by incorporating molecular data as critical components for guiding the diagnosis and treatment of CNS tumors.^2–4^

These developments combined with the rarity, disease-specific tumor biology, and clinical implications underscore the necessity for specialized training, especially as our understanding of these malignancies keeps evolving rapidly.

The European Society for Medical Oncology (ESMO) CNS Faculty aims at identifying the essential educational needs of professionals operating in the field of CNS oncology.

## Methods

The ESMO CNS Faculty promoted a consensus process, distributing a web-based questionnaire to ESMO Faculty members and other ESMO professionals active in the field of CNS oncology, ensuring balanced participation without regard to country or professional background. The objective was to identify and prioritize the most relevant educational needs of physicians treating CNS cancers. Responses were collected, analyzed, and categorized by frequency and perceived importance.

The questionnaire presented the following question, allowing a free-text response format: “In your opinion, what are the 3 most important educational needs for training clinical and/or research fellows in CNS oncology over the next 10 years?”

The survey was distributed online on February 28, 2023, to the current ESMO CNS Faculty group and to ESMO members selected based on interest and/or activity in neuro-oncology declared in the ESMO membership database. Data were collected and elaborated anonymously, and the results were presented and discussed during the last CNS Faculty group meeting at the ESMO 2024 Congress. The free-text answers were analyzed using an inductive content analysis approach performed independently by 2 investigators, with arbitration by a third reviewer when necessary. The coded responses were subsequently grouped into 8 overarching categories based on keyword convergence and thematic similarity. The categories were molecular and diagnostic tools in neuro-oncology (45 responses, 22%), translational and clinical research skills (27, 13%), multidisciplinary and multiprofessional care (28, 13%), supportive care (26, 13%), CNS management and treatment (24, 12%), neuro-oncology educational training (22, 11%), neuroimaging (19, 9%), and networking and career building (17, 8%). Frequencies were then calculated according to the number of mentions in each category.

Results of the survey are presented here in a consensus position paper.

## Results

### Study Population

A total of 318 physicians received the questionnaire, and 80 (25%), from 24 different countries across the globe, replied to the survey. A total of 208 answers were collected from all participants in the top 3 needs for CNS education.

**Figure 1.**
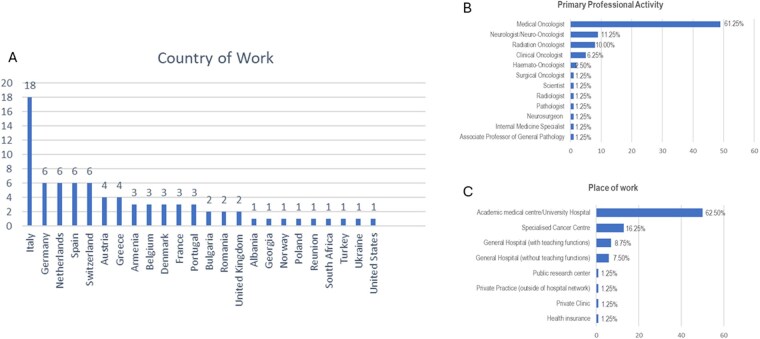
Country of work (A), primary professional activity (B), and place of work (C) of the participants in the ESMO CNS Faculty Survey. CNS, Central Nervous System; ESMO, European Society for Medical Oncology.

**Figure 2.**
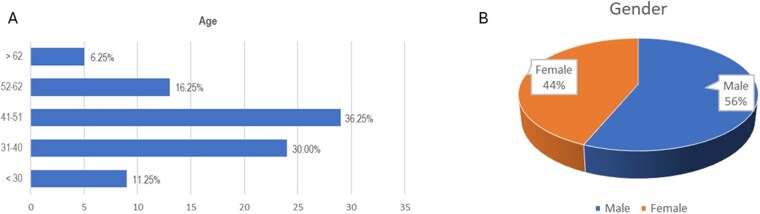
Age (A) and gender (B) of the participants in the ESMO CNS Faculty Survey. CNS, Central Nervous System; ESMO, European Society for Medical Oncology.

Most of the respondents work in Italy (22.5%), followed by Germany, Spain, Switzerland, and the Netherlands (7.5% for each country) (Figure 1A). Unsurprisingly, the majority of respondents were medical doctors (97.5%), with the following specializations (most frequently listed): medical oncologist (61.25%), neurologist/neuro-oncologist (11.25%), and radiation oncologist (10%). Only 1 neurosurgeon answered the survey (Figure 1B). One out of every 5 respondents did not work in an academic center/university hospital (62.5%) nor in a specialized cancer center (16.25%) (Figure 1C). Most respondents were relatively young, with the largest age groups 31-40 years old (30%) and 41-51 years (36.25%) (Figure 2A). Participants were similarly distributed between men (56%) and women (44%; *P* > .05) (Figure 2B).

**Figure 3.**
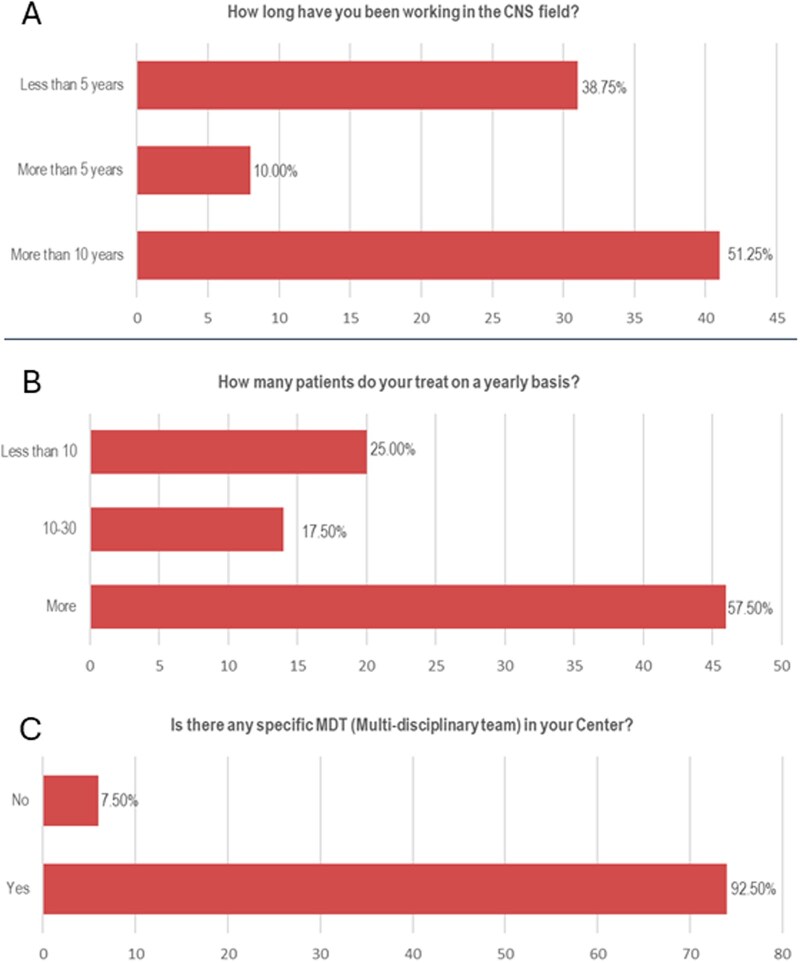
Years of experience in the CNS field (A), annual number of patients treated (B), and MDT in their centers (C) of the participants in the ESMO CNS Faculty Survey. CNS, Central Nervous System; ESMO, European Society for Medical Oncology; MDT, multidisciplinary team.

In terms of experience, 51.25% of respondents have more than 10 years of experience in neuro-oncology field, while 38.75% have less than 5 years of experience (Figure 3A). More than half of the responders (57.50%) treat more than 30 CNS tumor patients per year (Figure 3B). Almost all responders (92.5%) work in a center with a dedicated multidisciplinary team (MDT) (Figure 3C).

### Educational Needs Identified

**Figure 4.**
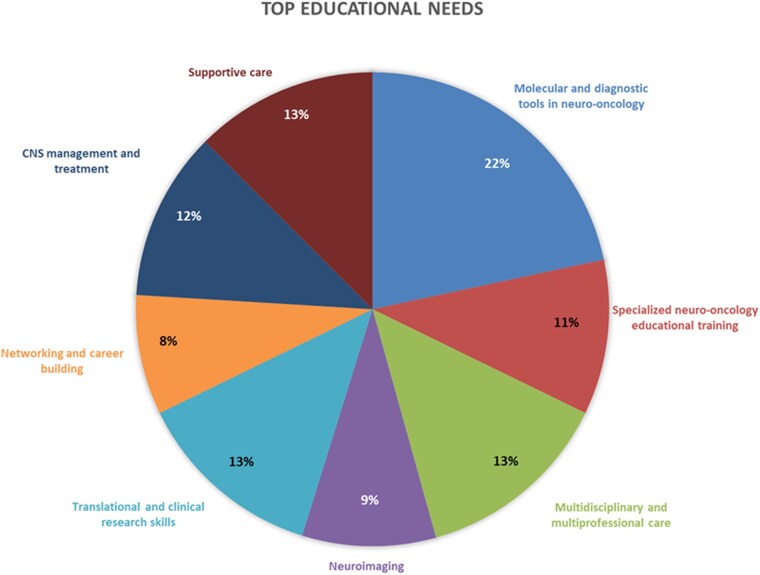
Pie chart of top educational needs by Participants in the ESMO CNS Faculty Survey. CNS, Central Nervous System; ESMO, European Society for Medical Oncology.

Based on the survey responses, we identified 8 categories and organized the expressed needs accordingly. These 8 categories, ordered by frequency (high to low) by our respondents, are listed below (Figure 4):

1. Molecular and diagnostic tools in neuro-oncology (45, 22%)
2. Translational and clinical research skills (27, 13%)
3. Multidisciplinary and multiprofessional care (28, 13%)
4. Supportive care (26, 13%)
5. CNS management and treatment (24, 12%)
6. Neuro-oncology educational training (22, 11%)
7. Neuroimaging (19, 9%)
8. Networking and career building (17, 8%)

Several categories extend beyond CNS oncology, reflecting general educational needs that clinicians treating CNS tumors consider essential for practice and ongoing professional development. This aligns with the ESMO CNS Faculty perspective that educational cornerstones are common across oncology disciplines, including rare cancers such as brain tumors.

#### Molecular and Diagnostic Tools in Neuro-Oncology

The classification of CNS tumors has advanced rapidly over the last 5 years, driven by new molecular and pathological insights, which have been incorporated into clinical practice following the 2021 WHO guidelines.^3^^,^^4^ For CNS oncologists, essential educational priorities regarding this matter include:

**Table:** 

Learning item	Description
Mastering the WHO classification:	✓ Understanding new tumor entities ✓ Understanding evolving nomenclature^5^^,^^6^
Acquiring comprehensive training in molecular diagnostics:	✓ Techniques and available assays with pros/cons ✓ Disease-specific molecular profiling and their prognostic/predictive value ✓ Clinical interpretation of results for evidence-based treatment decisions
Immuno-biology and microenvironment	✓ Unique interaction between immune system and CNS microenvironment

#### Translational and Clinical Research Skills

Clinical practice needs to include clinical research skills, allowing professionals to correctly interpret evidence and contribute to the design of new trials. Specific useful skills should include:

**Table:** 

Learning item	Description
Expertise in clinical trials:	✓ Research methodology ✓ Protocol design ✓ Data management and interpretation ✓ Gain practical experience in biostatistics
Experience in translational research:	✓ Organoids ✓ Patient-derived xenografts ✓ Liquid biopsy ✓ Cerebrospinal fluid (CSF) analysis

The survey results indicate that there is a strong emphasis on acquiring the ability to design trials specifically applicable to neuro-oncology, with a potential focus on innovative clinical trial design approaches such as platform trials, tumor-agnostic strategies, Phase 0 studies, and window-of-opportunity trials.

#### Multidisciplinary and Multiprofessional Care

Multidisciplinary and multiprofessional care are seen as key elements in the management of patients with cancer. In the case of neuro-oncology, this aspect needs to be taken even more into consideration. Specifically, ESMO members feel that particular attention should be given to:

**Table:** 

Learning item	Description
MDT including:	✓ Understanding the process by which and the conditions under which expertise diversity may promote team performance. ✓ Medical oncologists ✓ Radiation oncologists ✓ Neurologists ✓ Neurosurgeons ✓ Neuro-pathologists ✓ Neuro-radiologists ✓ Nuclear medicine specialists ✓ Main goal: Optimize treatment
Healthcare professionals in neuro-oncology should:	✓ Have specific expertise in neuroanatomy ✓ Be proficient in neurological clinical evaluation and be able to identify differential diagnoses and avoid potentially detrimental use of steroids ✓ Management of acute and chronic complications, such as seizures, brain edema, neurological symptoms ✓ Knowledge regarding treatment side effects, including steroids induced toxicity, anti-seizure medication induced toxicity immuno-
	neurological toxicities (eg CAR-T related), management of intrathecal therapy toxicity✓ Gaining expertise in recognizing and managing neurological emergencies✓ Solid understanding of neurophysiology
Training in cognitive and neurological function assessment:	✓ Monitoring impairments and cognitive function and when to address patients to neurological rehabilitation
Training in radiotherapy	✓ Direct experience in the radiotherapy department ✓ Exploring benefits from combinations of systemic treatment and radiotherapy
CNS metastases	✓ Training in multidisciplinary management of brain metastases and leptomeningeal metastases ✓ Toxicity deriving from radiotherapy combined with systemic treatments ✓ Administration of intrathecal therapy

#### Supportive Care

Supportive care is a cornerstone in neuro-oncology, since maintaining quality of life remains a central concern for patients, who may endure both disease-related deficits and therapy-related toxicities. According to respondents, learning priorities in supportive care include:

**Table:** 

Learning item	Description
Effective management of neurological supportive treatment:	✓ Use of steroids ✓ Use of anti-epileptic medications
Activation of rehabilitation programs to address disease-specific challenges:	✓ Rehabilitative interventions for cognitive impairment, language disorders, and physical deficits. ✓ Managing patients with mobility impairment or movement disorders ✓ Managing psychological and psychiatric needs of patients ✓ Maintain good performance status
Planning supportive and palliative care:	✓ Knowledge of regional landscape of palliative care organizations ✓ Communication with patients and families ✓ End of life care management and timing

#### CNS Management and Treatment

A few aspects of neuro-oncology require focused attention, particularly gray areas in treatment strategies and options. Key training priorities for CNS oncologists include:

**Table:** 

Learning item	Description
Innovative treatments in glioma	✓ Management of glioma after progression to first-line therapy ✓ Optimizing the knowledge on the role of novel therapies (targeted agents, immunotherapy, IDH inhibitors, and tumor-treating fields), as well as guidance on accessing these therapies and clinical trials ✓ Integration of molecular data into evidence-based treatment planning ✓ Applying the best therapeutic approaches to low-grade gliomas and other CNS tumors.
Rare histologies	Acquiring proper training in managing rare histologies

#### Specialized Neuro-Oncology Educational Training

Availability of useful training resources and proper clinical training represent a key step to acquire all necessary skills for a neuro-oncologist, including the management of neurological symptoms and signs associated with brain tumors and the identification of differential diagnoses. Participants also underline the disparity and differences among countries in terms of drug availability and professional figures caring for these patients; this is a critical aspect that might be improved by more accessibility and diffusion of standardized guidelines.

This aspect specifically includes:

**Table:** 

Learning item	Description
Participation in preceptorships and workshops:	✓ Active clinical case discussions ✓ ESMO preceptorships ✓ EANO school for Neuro-Oncology
Access to disease guidelines	✓ EANO ✓ ESMO ✓ ESTRO ✓ SNO ✓ ASCO ✓ Local guidelines
Acquire direct clinical expertise	✓ Rotation in a center of excellence ✓ Rotation in neurology for medical oncologists and rotation in oncology for neurologists ✓ Being more actively involved during training ✓ Participation in multidisciplinary CNS tumors boards
Acquire pediatric experience	✓ Not only training with adults, but also with pediatrics due to the particularity of the field

#### Neuroimaging

Radiological diagnosis is a fundamental step in identifying CNS tumors. Participants recognize the importance of learning about neuroimaging in both diagnostic setting and tumor response assessment while also emphasizing the significance of research opportunities in neuroimaging.

**Table:** 

Learning item	Description
Neuroimaging	✓ CNS tumor diagnosis vs common differential diagnosis ✓ Tumor response assessment ✓ Pseudoprogression/real tumor progression ✓ Radionecrosis ✓ Tumor or therapy induced complications
Advanced neuroimaging	✓ Clinical indications for advanced imaging ✓ Neuronal network analysis ✓ Radiomics and machine learning applications
Nuclear medicine	✓ Amino acid PET scans and tracers

#### Networking and Career Building

Neuro-oncology education should acknowledge the challenges of managing rare tumors in smaller centers; thus, referring to expert centers is recommended. Given the rarity of these tumors, active participation in national and international collaborative networks and societies is also essential, complementing existing efforts.

The survey revealed that networking is seen as one of the most critical needs in neuro-oncology, specifically in terms of:

**Table:** 

Learning item	Description
Networking in clinical practice	✓ Collaboration with international experts ✓ Exchange of ideas ✓ Collaboration with high volume centers ✓ Granting trials access through international networks ✓ Establishing networks and meetings aiming at reducing heterogeneity in diagnosis and treatment ✓ Addressing disparity in terms of drug availability and experts caring for these patients ✓ Involvement of patient’s representatives
Networking in research	✓ High quality cancer registries to enhance data surveillance and monitoring ✓ Attending conferences supported by grants, independently from pharmaceutical support ✓ Increasing enthusiasm for neuro-oncology. ✓ Involvement of patient’s representatives

Participants also emphasized the need for standardized guidelines to simplify and optimize patient referrals to specialized centers, particularly for clinical trial enrollment. Furthermore, many emphasized the importance of fostering interdisciplinary connections between basic and clinical research to drive advancements in translational research for CNS tumors.

## Discussion

The ESMO CNS Faculty, in order to pursue ESMO’s educational mission, worked on a consensus paper to investigate and summarize the educational needs of trainees according to CNS oncologists.

A total of 80 members, mainly from 31 to 51 years old, working in the field of neuro-oncology answered the survey. The majority work as medical oncologists in different European countries, especially in academic medical centers and specialized cancer centers, with direct experience working with MDTs. However, the response rate was low (25%), and the sample was predominantly European, with the largest contributions from Italy, Germany, and Spain and minimal participation from low- and middle-income countries or non-European regions. These features introduce potential selection and nonresponse bias, likely over-representing clinicians embedded in academic or highly networked centers, and limit the generalizability of the findings. Given that most respondents were medical oncologists (62.5%), as the survey was led by the ESMO society, this distribution may have influenced the prominence of general oncology domains within the results. Although respondents represented a range of experience levels, and >50% of respondents had >10 years of experience in the neuro-oncology field, we did not perform stratified analyses by years of CNS practice; future surveys should assess whether educational priorities differ between early career and highly experienced neuro-oncology professionals.

Answers led to the identification of 8 different categories of educational needs classified by frequency mentioned by participants (Figure 4). Respondents expressed a primary interest in collaborating within national and international connected teams and networks to maximize knowledge and optimize therapeutic strategies available in neuro-oncology. They emphasized the importance of maintaining a focus on the new opportunities that clinical and basic science research can offer.

High relevance has been attributed to adequate training in pathology and molecular diagnostics, especially regarding clinical implications. A survey performed by the Society for Neuro-oncology (SNO) in 2021,^7^ demonstrates that most neuro-oncology physicians are aware of recent developments according to diagnostic pathways reflecting the shift in integrated molecular and histopathologic classification reflected in the updated 2021 WHO diagnostic criteria. However, the consequences of these developments in daily clinical practice increase the need for applied educational programs, in accordance with our survey.

Similarly, respondents perceive multidisciplinary teamwork as a key feature in neuro-oncology training because care encompassing all domains of life requires intensive collaboration between physicians, other healthcare professionals, patients, and caregivers, as the survey confirms.^8^ Clinical trials represent the highest standard and quality of care in medicine and, especially in neuro-oncology, where therapeutic possibilities are limited, are of utmost importance; unfortunately, access remains unequal for various reasons.^9^ CNS clinicians demonstrated high sensibility to equity in CNS cancer care, trial access, and supportive care; it is recognized that efforts are needed to set universal standards and therefore guarantee the best possible care at every step of the treatment process. Specialized neuro-oncology educational training emerged as another key priority, including increasing society-led preceptorships, easy access to international and local guidelines, rotations in centers of excellence, and multidisciplinary CNS tumor boards. To address jurisdictional boundaries between adult and pediatric practice and in line with respondents’ suggestion to “acquire pediatric experience,” this should be addressed via supervised exposure, such as joint tumor boards, observerships, and shared modules across scientific societies and specialties involved in CNS tumor care, rather than cross-licensure, thereby strengthening competency while respecting national regulations.

The need to be specifically trained in communicating effectively with patients and families about supportive care, palliative care, and end-of-life issues was highlighted in our survey as a priority among CNS specialists. It is well known that people suffering from brain tumors have profound supportive care and rehabilitation needs, extending to their families, who benefit from psychological support and guidance. However, inequality in the delivery of such care persists, often due to limited specialized services, long waiting lists, and insufficient staff of health professionals.^10^ Notably, a 2017 SNO survey of 29 adult neuro-oncology fellowship programs recognized the need to train fellows in palliative and end-of-life care and reported active efforts to incorporate such training into standard curriculum.^11^ Our survey confirms that this gap remains and underscores the need to strengthen CNS-specific education programs.

Based on the survey results, we outline concrete actions: (1) update the ESMO CNS educational book with molecular, competency-based content; (2) perform SWOT analyses for neuro-oncology training and practice in each member country; and (3) promote area-specific educational webinars, preceptorships, and include podcasts to increase the CNS knowledge broadcast. These steps will leverage existing society infrastructures to standardize training and promote equitable access to CNS oncology expertise across ESMO, EANO, and SNO.

ESMO is well equipped to participate in the education of CNS tumor specialists together with other societies, such as EANO and SNO, through specific neuro-oncology preceptorships and broader initiatives related to curriculum building and mentoring.

## Data Availability

The dataset generated and analyzed in this study consists of anonymized survey free-text responses collected from physicians through an ESMO CNS Faculty-led questionnaire; it contains no patient-level or clinical data. Data can be shared upon reasonable request to the corresponding author, subject to approval by the ESMO CNS Faculty/ESMO (as data custodian) and, when required, a data-sharing agreement ensuring that confidentiality and anonymity are preserved.
